# Supplementary material for: Persistently Elevated HBV Viral-Host Junction DNA in Urine as a Biomarker for Hepatocellular Carcinoma Minimum Residual Disease and Recurrence: A Pilot Study
Source: Diagnostics (Basel). 2023 Apr 25;13(9):1537. doi: 10.3390/diagnostics13091537 (PMC10177231; doi:10.3390/diagnostics13091537)
Supplement: Supplementary file 1 [file diagnostics-13-01537-s001.zip › diagnostics-2249829-supplementary.pdf]

SUPPLEMENTARY FIGURES

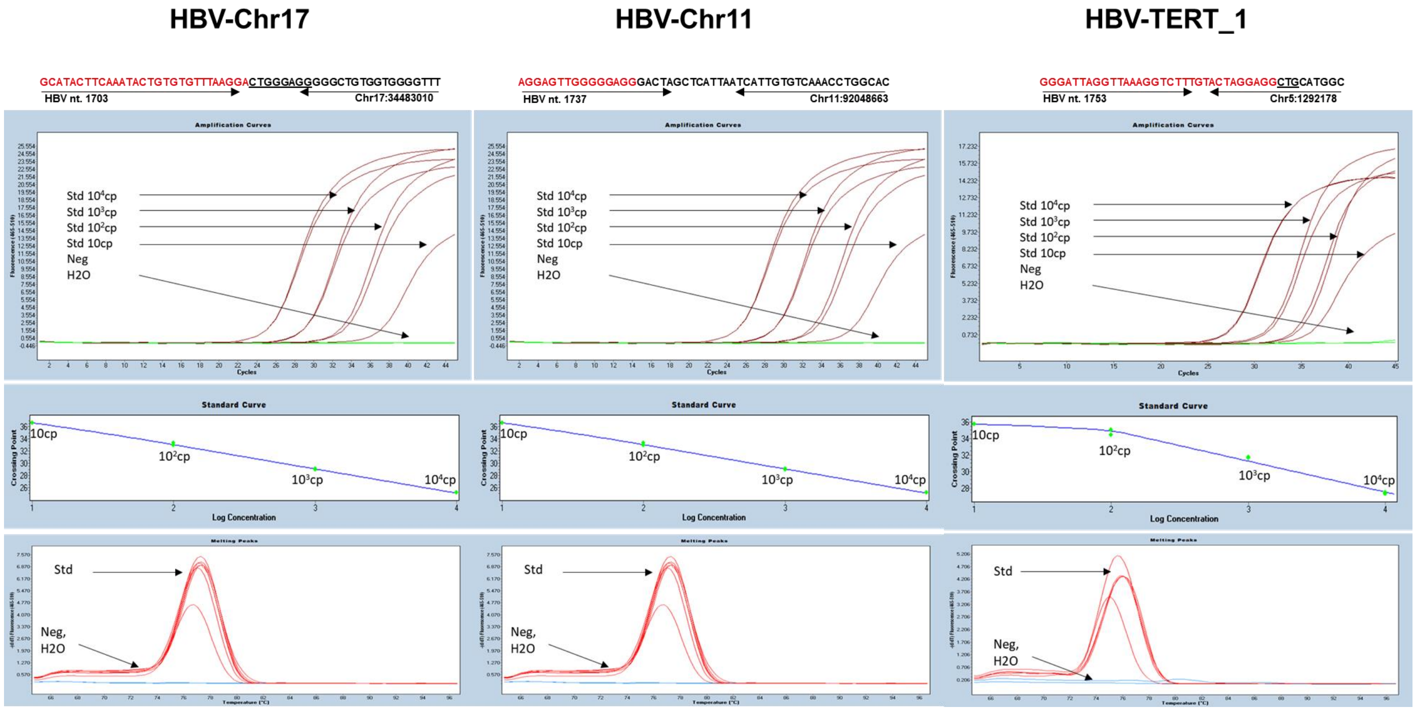

Supplemental Figure S1. Development of short amplicon HBV-JS quantitative PCRs. Amplicon sequences and their start and end coordinates are listed for each junction sequence. The HBV DNA sequences are in red and human DNA sequences are in black, and black underlined sequences indicate overlap between HBV and human sequences. Each quantitative assay was developed using junction-specific positive DNA control as a standard (Std) and normal human DNA as a negative control (Neg). Amplification, standard, and melting curves are shown for each HBV-JS biomarker.

SUPPLEMENTARY TABLES

Supplemental Table S1. HBV-JS qPCR primers used.

Assay (amplicon size, bp)	Patient Case #	5' -3'Sequence	PCR Annealing Tm^
HBV-Chr17 (54)	1	GCATACTTCAAATACTGTGTGTTTAAGG	65°C
		AAAACCCCACCACAGCCCCC	
HBV-Chr11 (50)	2	AGGAGTTGGGGGAGGGACTA	65°C
		GTGCCAGGTTTGACACAATGA	
HBV-TERT_1 (41)	3	GCCATGCAGCCTCCTAGT	60°C
		GGGAGGAGATTAGGTTAAWGGTCTT	

W is a degenerate base (A/T); ^, Quantitative PCR was performed with Roche's LightCycler® 480 platform using LightCycler® 480 SYBR Green I Master with a final 1μM primer concentration per PCR reaction.
